# Consumption of Green Tea, but Not Black Tea or Coffee, Is Associated with Reduced Risk of Cognitive Decline

**DOI:** 10.1371/journal.pone.0096013

**Published:** 2014-05-14

**Authors:** Moeko Noguchi-Shinohara, Sohshi Yuki, Chiaki Dohmoto, Yoshihisa Ikeda, Miharu Samuraki, Kazuo Iwasa, Masami Yokogawa, Kimiko Asai, Kiyonobu Komai, Hiroyuki Nakamura, Masahito Yamada

**Affiliations:** 1 Department of Neurology and Neurobiology of Aging, Kanazawa University Graduate School of Medical Sciences, Kanazawa, Japan; 2 Department of Physical Therapy, Division of Health Sciences, Kanazawa University Graduate School of Medical Sciences, Kanazawa, Japan; 3 Bishoen Geriatric Health Services Facility, Suzu, Japan; 4 Department of Neurology, Ioh Hospital, National Hospital Organization, Kanazawa, Japan; 5 Department of Environmental and Preventive Medicine, Kanazawa University Graduate School of Medical Sciences, Kanazawa, Japan; Cardiff University, United Kingdom

## Abstract

Our objective was to determine whether the consumption of green tea, coffee, or black tea influences the incidence of dementia and mild cognitive impairment (MCI) in older people. We conducted a population-based prospective study with Japanese residents aged >60 years from Nakajima, Japan (the Nakajima Project). Participants received an evaluation of cognitive function and blood tests. The consumption of green tea, coffee, and black tea was also evaluated at baseline. Of 723 participants with normal cognitive function at a baseline survey (2007–2008), 490 completed the follow up survey in 2011–2013. The incidence of dementia during the follow-up period (mean ± SD: 4.9±0.9 years) was 5.3%, and that of MCI was 13.1%. The multiple-adjusted odds ratio for the incidence of overall cognitive decline (dementia or MCI) was 0.32 (95% CI: 0.16–0.64) among individuals who consumed green tea every day and 0.47 (95% CI: 0.25–0.86) among those who consumed green tea 1–6 days per week compared with individuals who did not consume green tea at all. The multiple-adjusted odds ratio for the incidence of dementia was 0.26 (95% CI: 0.06–1.06) among individuals who consumed green tea every day compared with those who did not consume green tea at all. No association was found between coffee or black tea consumption and the incidence of dementia or MCI. Our results indicate that green tea consumption is significantly associated with reduced risk of cognitive decline, even after adjustment for possible confounding factors.

## Introduction

Coffee and tea are widely consumed around the world. In Japan and other Asian countries, green tea is a popular beverage, whereas in the Western countries, black tea is popular. Coffee, tea and tea-related polyphenols have been extensively studied for their neuroprotective effects and their potential for preventing neurodegenerative diseases, including Alzheimer's disease (AD) [1]–[7]. Coffee and tea contain large amounts of caffeine, which has been investigated for its neuroprotective effects both *in vivo* and *in vitro* [8], [9]. However, evidence from cohort studies that examine the relationship between green tea or coffee consumption and dementia is limited and inconsistent.

Several longitudinal studies [10]–[13] have investigated the relationship between coffee consumption and dementia, AD, or cognitive decline, but findings from these studies are also inconsistent. In addition, longitudinal studies of black tea consumption have not found any association with reduced risks for dementia, AD, or cognitive decline [14], [15]. One cross-sectional study has shown that higher green tea consumption is associated with lower prevalence of cognitive impairment [16].

We hypothesized that the consumption of beverages rich in polyphenols and caffeine, such as green tea, coffee, or black tea, would be protective and would delay the onset of dementias including AD. In the present longitudinal study, we aimed to determine whether the consumption of the aforementioned beverages is associated with the incidence of dementia and mild cognitive impairment (MCI) in the general population.

## Methods

### Study participants

The Nakajima Project was a population-based cohort study that investigated correlations between lifestyle and the prevalence of dementia in elderly Japanese individuals. The study was conducted in Nakajima, in the Nanao district of Ishikawa Prefecture, Japan. The study design was described previously [17], [18].

Participants were recruited as a part of the Nakajima Project. The baseline survey was conducted between 2007 and 2008. On April 1, 2007, 2,845 people who were 60 years or older were legally residing in Nakajima. These elderly residents were eligible to receive a free evaluation of their physical health, which included a battery of neurological and cognitive tests that examined cognitive function. Recruitment began in May 2007 by distributing flyers to Nakajima residents. Nakajima project was supported by Nanao city, and the information of the residence was used to list target candidates. The baseline survey included questionnaires regarding personal lifestyle, medical conditions, and neuropsychological tests. All participants lived in the community at the time of the baseline survey. Blood samples were also collected, and ascorbic acid (vitamin C) levels and ApoE phenotypes were determined for all participants. The study was conducted by 14 neurologists, two psychologists, seven nurses, one physiotherapist, and one occupational therapist, all of whom were specifically trained for this study.

### Baseline survey

Each participant completed a self-administered questionnaire that queried sociodemographic data (including age, sex, and education), past medical history (hypertension, hyperlipidemia, and diabetes mellitus), smoking habits, physical activities/hobbies, and green tea, coffee, and black tea consumption. The trained researchers reviewed the completed questionnaires to identify inconsistent or unanswered items. Green tea, coffee, and black tea consumption was quantified by the frequency of consumption of each beverage using the choice of 0, 1, 2, 3, 4, 5, or 6 times/week or every day. For the present analysis, we further divided this category into three groups: zero (no consumption), 1–6 days/week, and every day.

We assessed participants' cognitive status with the Mini-Mental State Examination (MMSE) [19] and the Clinical Dementia Rating (CDR) [20]–[22]. Higher MMSE scores indicate higher cognitive function, and the maximum score is 30 points. Standard cut point of <24 out of 30 indicates cognitive impairment [19]. CDR is a dementia-staging device that rates cognitive function from none to maximal along five levels of impairment (rated as 0, 0.5, 1, 2, or 3) in each of the following six domains: memory, orientation, judgment and problem solving, function in community affairs, home and hobbies, and personal care. A global CDR score was calculated using an algorithm that takes into account each of these domain subscores. The possible scores for global CDR were 0 (indicating a normal healthy individual with no cognitive or functional deficits), 0.5 (a normal healthy individual with questionable cognitive and/or functional abilities), 1 (mild dementia), 2 (moderate dementia), and 3 (severe dementia).

High-performance liquid chromatography (HPLC) was used to measure total serum ascorbic acid concentrations [23]. ApoE phenotype was determined using isoelectric electrophoresis as described by Kamboh et al [24].

Diagnosis of dementia was based on the guidelines of the Diagnostic and Statistical Manual of Mental Disorders, Third Edition, Revised (DSM-III-R) [25]. Diagnosis of MCI was established according to the International Working Group on general criteria for MCI [26]. The MCI criteria state that (1) a person should be judged as abnormal using modalities other than those used to fulfill the DSM-III-R dementia criteria, (2) the person's functional activities are mainly preserved or at least impairment is minimal, and (3) the person should have evidence of cognitive decline, either by self-assessment and/or by an informant report in conjunction with deficits in objective cognitive tasks. Among participants without dementia, a CDR score of 0.5 was used as the objective cognitive impairment value to denote cognitive and functional impairment consistent with MCI.

### Follow-up survey

During 2011 and 2013, all subjects who could be contacted and who agreed to participate in the follow-up survey were interviewed to determine whether their health status or cognitive functioning had changed. The follow-up survey was conducted in public halls or at the participants' homes. Participants completed the same questionnaires and underwent the same neurological tests that were administered at the initial baseline survey. In addition, we visited seven individuals who were institutionalized in long-term care facilities or hospitals, and administered the same examinations after obtaining written informed consent from their families. Participants who died or who left Nakajima before the follow-up was conducted were excluded from the analysis, and the date they died or moved away were obtained from Nanao city.

### Standard protocol approvals and patient consents

This study was conducted with the approval of the medical ethics review board of Kanazawa University (Kanazawa, Japan). All participants provided written informed consent by signing a form that described the purpose and procedures of the study, the potential risks and benefits associated with participation, the strict voluntary nature of participation, the right to withdraw from the research without prejudice or penalty, and a guarantee of confidentiality and security of personal data.

### Statistical analyses

Baseline characteristics were evaluated using a one-way ANOVA for continuous variables and the chi-square test for categorical variables. Trend tests were conducted for green tea, coffee, and black tea consumption to test the significance of these variables. Univariate and multivariate logistic regression models were used to analyze the independent effects of green tea, coffee, and black tea consumption on the risk of developing dementia or MCI so that the lowest category served as the reference group. Model 1 was sex- and age- adjusted. Model 2 was further adjusted for history of hypertension, diabetes mellitus, and hyperlipidemia, formal education, and ApoE phenotype status (ApoE E4+ or E4−). Model 3 was fully adjusted and included smoking status, alcohol consumption, green tea, coffee, and/or black tea consumption, physical activities and/or hobbies. A two-sided P-value less than 0.05 was considered statistically significant in all analyses. The SPSS software package (version 12.0J; SPSS Inc., Chicago, IL) was used to perform all statistical analyses.

## Results

Of the 2,845 potential candidates, 982 voluntarily participated in the brain-function examination conducted at public town halls in 2007–2008 (participation rate: 34.5%). We excluded 217 subjects from the analysis because of dementia (n = 8), MCI (n = 205), or failure to complete the cognitive tests (n = 4). We further excluded 42 subjects with a MMSE score of <24 at the initial baseline survey. Thus, 723 participants were judged to have normal cognitive function at the initial baseline survey. Of the 723 participants with normal cognitive function, 55 died, 5 moved, 167 did not repeat cognitive testing at follow-up, and 6 incompletely answered the beverage-frequency questionnaire at the initial baseline survey. Thus, data from 490 participants were included in the final analysis. The sociodemographic characteristics between participants included in the final analysis and subjects lost to follow-up did not differ in age, MMSE scores, or formal education years at the time of the baseline survey (Table S1).

With regard to green tea, 195 participants (39.8%) drank moderate amounts (1–6 days per week), 157 (32.0%) drank every day, and 138 (28.2%) did not drink green tea. With regard to coffee intake, 212 participants (43.3%) drank coffee every day, 180 (36.7%) drank moderate amounts (1–6 days per week), and 98 (20.0%) did not drink coffee. As for black tea consumption, 404 participants (82.4%) did not consume, whereas 86 (17.6%) consumed black tea at least 1 day per week. Because the number of participants drinking black tea for every day was too small (n = 6), we grouped together the participants drinking black tea for 1–6 days/week and every day.

Tables 1–3 show results from the baseline and follow-up surveys according to green tea, coffee, and black tea consumption categories. At baseline, participants who consumed larger amounts of green tea was younger (P<0.001), had more formal education (P<0.001), had higher MMSE scores (P<0.001), and had higher scores for current physical activities and/or hobbies (P = 0.008). More frequent consumption of coffee was associated with lower age (P<0.001) and higher ascorbic acid levels (P = 0.008) at baseline along with higher scores for current physical activities and/or hobbies (P = 0.02). Participants who did not consume black tea had fewer years of formal education (P = 0.005) and tended to have lower MMSE scores (P = 0.083) than those who consumed black tea more than 1 day per week.

**Table 1:** Characteristics at baseline survey of the participants according to green tea, coffee, and black tea consumption (part 1).

	Green tea				Coffee				Black tea		
Beverage consumption, days/week	None	1–6 days/week	Every day	P-value (trend)	None	1–6 days/week	Every day	P-value (trend)	None	1–7 days/week	P-value
Number of subjects	n = 138	n = 195	n = 157		n = 98	n = 180	n = 212		n = 404	n = 86	
Age at baseline survey, years	73.1 (7.2)	70.0 (5.7)	71.0 (6.1)	<0.001	73.4 (5.8)	71.1 (6.5)	70.2 (6.3)	<0.001	71.4 (6.5)	70.3 (6.0)	0.225
Sex: women, %	70.3	62.1	70.1	0.972	62.2	65.6	70.3	0.140	65.8	72.1	0.264
Education, years	9.1 (2.1)	9.9 (2.0)	10.5 (2.3)	<0.001	9.6 (2.1)	9.9 (2.3)	10.0 (2.2)	0.294	9.6 (2.2)	10.5 (2.4)	0.005
MMSE, points, Median (SE)	27.0 (0.2)	29.0 (0.1)	29.0 (0.1)	<0.001	28.0 (0.2)	28.5 (0.1)	28.0 (0.1)	0.131	28.0 (0.1)	29.0 (0.2)	0.083
ApoE E4 carriers, %	17.5	23.6	23.5	0.227	20.6	19.4	24.4	0.344	21.8	22.1	0.948
HT at baseline, %	39.9	44.1	48.4	0.140	45.9	46.1	42.0	0.439	43.8	46.5	0.648
HL at baseline, %	18.1	16.9	16.6	0.727	17.3	17.8	16.5	0.808	16.8	18.6	0.692
DM at baseline, %	9.4	12.8	15.3	0.132	13.3	11.7	13.2	0.918	12.6	12.8	0.966

Values expressed as mean (SD) unless otherwise indicated.

MMSE: Mini-mental state examination, HT: hypertension, HL: hyperlipidemia, DM: diabetes mellitus.

**Table 2:** Characteristics at baseline survey of the participants according to green tea, coffee, and black tea consumption (part 2).

	Green tea				Coffee				Black tea		
Beverage consumption, days/week	None	1–6 days/week	Every day	P-value (trend)	None	1–6 days/week	Every day	P-value (trend)	None	1–7 days/week	P-value
Number of subjects	n = 138	n = 195	n = 157		n = 98	n = 180	n = 212		n = 404	n = 86	
Smokers (Current), %	11.6	13.3	7.0	0.186	9.2	8.3	13.7	0.143	11.6	7.0	0.207
Alcohol drinkers (Current), %	34.1	42.1	35.7	0.826	32.7	42.2	36.3	0.810	38.6	33.7	0.396
Physical activities/hobbies (Current), %	67.4	76.9	80.9	0.008	71.4	70.6	81.6	0.02	75.0	77.9	0.570
Green tea consumption,	-	-	-	-	30.6/34.7	43.3/32.2	41.0/30.7	0.965	37.9/30.9	48.8/37.2	0.011
1–6 days/everyday, %											
Coffee consumption,	31.9/43.5	40.0/44.6	36.9/41.4	0.965	-	-	-	-	33.9/44.6	50.0/37.2	0.877
1–6 days/everyday, %											
Black tea consumption,	8.7	21.5	20.4	0.011	11.2	23.9	15.1	0.877	-	-	-
1–7 days/week, %											
Ascorbic acid, µg/mL	6.8 (3.4)	6.8 (3.5)	6.9 (3.2)	0.834	6.0 (3.2)	6.7 (3.2)	7.3 (3.5)	0.008	6.7 (3.3)	7.3 (3.6)	0.147

Values expressed as mean (SD) unless otherwise indicated.

**Table 3:** Characteristics at follow-up survey of the participants according to green tea, coffee, and black tea consumption.

	Green tea				Coffee				Black tea		
Beverage consumption, days/week	None	1–6 days/week	Every day	P-value (trend)	None	1–6 days/week	Every day	P-value (trend)	None	1–7 days/week	P-value
Number of subjects	n = 138	n = 195	n = 157		n = 98	n = 180	n = 212		n = 404	n = 86	
Age at follow-up survey, years	78.0 (7.1)	75.0 (5.8)	75.8 (6.1)	0.001	78.4 (5.9)	76.1 (6.6)	75.1 (6.3)	<0.001	76.3 (6.5)	75.3 (6.0)	0.251
Follow-up time, years	4.9 (0.8)	5.0 (0.8)	4.8 (1.0)	0.333	4.9 (0.8)	4.9 (0.8)	4.9 (0.9)	0.963	4.9 (0.8)	4.9 (0.9)	0.940
MMSE, points, Median (SE)	27.0 (0.3)	28.5 (0.2)	29.0 (0.2)	0.001	27.0 (0.3)	28.0 (0.3)	29.0 (0.2)	0.016	28.0 (0.2)	29.0 (0.3)	0.543
ΔMMSE (baseline-follow up), points	−0.95 (3.3)	−0.27 (2.7)	−0.46 (2.3)	0.295	−0.77 (2.6)	−0.64 (3.2)	−0.29 (2.4)	0.320	−0.48 (2.8)	−0.67 (2.9)	0.467
Dementia, N (%)	12 (8.7)	11 (5.6)	3 (1.9)	0.009	7 (7.1)	11 (6.1)	8 (3.8)	0.181	20 (5.0)	6 (7.0)	0.430
MCI, N (%)	31 (22.5)	18 (9.2)	15 (9.6)	0.001	13 (13.3)	23 (12.8)	28 (13.2)	0.985	54 (13.4)	10 (11.6)	0.728

Values expressed as mean (SD) unless otherwise indicated.

MMSE: Mini-mental state examination, MCI: mild cognitive impairment.

During the follow-up survey (follow-up period, 4.9±0.9 years), we documented 26 participants with dementia and 64 participants with MCI. Participants who consumed larger amounts of green tea were younger (P = 0.001), and had higher MMSE scores (P = 0.001). More frequent consumption of coffee was associated with lower age (P<0.001) and higher MMSE scores (P = 0.016). As shown in Tables 1–3, more frequent consumption of green tea was associated with lower incidence of dementia and MCI (P = 0.009 and P = 0.001, respectively). In contrast, no association was found between the frequency of coffee or black tea consumption and the incidence of dementia or MCI.

The relationships between green tea consumption and the incidence of cognitive decline (dementia or MCI) are shown in Table 4. We found that green tea consumption was inversely associated with the incidence of dementia in both age- and sex-adjusted models. With regard to the incidence of dementia, multivariate odds ratios were 1.00 (reference) for not consuming green tea, 0.90 (95% CI: 0.34–2.35) (P = 0.826) for consuming green tea 1–6 days per week, and 0.26 (0.06–1.06) (P = 0.06) for consuming green tea every day. Regarding the incidence of cognitive decline (dementia or MCI), multivariate odds ratios were 1.00 (reference) for not consuming green tea, 0.47 (0.25–0.86) (P = 0.015) for consuming green tea 1–6 days per week, and 0.32 (0.16–0.64) (P = 0.001) for consuming green tea every day (Table 4).

**Table 4:** Association between green tea consumption and the incidence of dementia or cognitive decline (MCI or dementia).

Frequency of green tea consumption	None	1–6 days/week	Every day
Dementia			
Number of cases	12	11	3
Unadjusted Model	1	0.64 (0.27–1.49)	0.21 (0.06–0.76)*
Model 1†	1	0.89 (0.36–2.19)	0.26 (0.07–0.94)*
Model 2§	1	0.89 (0.35–2.28)	0.27 (0.07–1.07)
Model 3¶	1	0.90 (0.34–2.35)	0.26 (0.06–1.06)
Cognitive decline (MCI or dementia)			
Number of cases	43	29	18
Unadjusted Model	1	0.39 (0.23–0.67)**	0.29 (0.16–0.54)***
Model 1†	1	0.53 (0.30–0.93)*	0.34 (0.18–0.64)**
Model 2§	1	0.49 (0.27–0.89)*	0.33(0.17–0.66)**
Model 3¶	1	0.47 (0.25–0.86)*	0.32 (0.16–0.64)**

Values expressed as odds ratios (95% CI) unless otherwise indicated.

*P-value <0.05.

**P-value <0.01.

***P-value <0.001.

† Model 1 was adjusted for age and sex.

§ Model 2 was adjusted as for model 1 plus history of hypertension, diabetes mellitus, hyperlipidemia, education, and ApoE E4 carrier status.

¶ Model 3 was adjusted as for model 2 plus alcohol drinking, smoking, physical activities and/or hobbies, and coffee and black tea consumption.

In contrast, we observed no association between coffee or black tea consumption and the incidences of either dementia or cognitive decline (Tables 5 and 6).

**Table 5:** Association between coffee consumption and the incidence of dementia or cognitive decline (MCI or dementia).

Frequency of coffee consumption	None	1–6 days/week	Every day
Dementia			
Number of cases	7	11	8
Unadjusted Model	1	0.86 (0.31–2.30)	0.51 (0.18–1.45)
Model 1†	1	1.06 (0.39–2.90)	0.69 (0.23–2.01)
Model 2§	1	1.13 (0.40–3.21)	0.71 (0.23–2.16)
Model 3¶	1	1.00 (0.34–2.99)	0.70 (0.22–2.17)
Cognitive decline (MCI or dementia)			
Number of cases	20	34	36
Unadjusted Model	1	0.93 (0.50–1.72)	0.80 (0.44–1.47)
Model 1†	1	1.22 (0.63–2.36)	1.19 (0.62–2.28)
Model 2§	1	1.23 (0.63–2.41)	1.09 (0.56–2.14)
Model 3¶	1	1.26 (0.62–2.54)	1.16 (0.58–2.32)

Values expressed as odds ratios (95% CI) unless otherwise indicated.

† Model 1 was adjusted for age and sex.

§ Model 2 was adjusted as for model 1 plus history of hypertension, diabetes mellitus, hyperlipidemia, education, and ApoE E4 carrier status.

¶ Model 3 was adjusted as for model 2 plus alcohol drinking, smoking, physical activities and/or hobbies, and coffee and black tea consumption.

**Table 6:** Association between black tea consumption and the incidence of dementia or cognitive decline (MCI or dementia).

Frequency of black tea consumption	None	1–7 days/week
Dementia		
Number of cases	20	6
Unadjusted Model	1	1.41 (0.55–3.61)
Model 1†	1	1.70 (0.64–4.47)
Model 2§	1	2.06 (0.76–5.61)
Model 3¶	1	2.14 (0.75–6.08)
Cognitive decline (MCI or dementia)		
Number of cases	74	16
Unadjusted Model	1	0.99 (0.54–1.81)
Model 1†	1	1.19 (0.64–2.24)
Model 2§	1	1.39 (0.72–2.68)
Model 3¶	1	1.52 (0.77–3.03)

Values expressed as odds ratios (95% CI) unless otherwise indicated.

† Model 1 was adjusted for age and sex.

§ Model 2 was adjusted as for model 1 plus history of hypertension, diabetes mellitus, hyperlipidemia, education, MMSE score, and ApoE E4 carrier status.

¶ Model 3 was adjusted as for model 2 plus alcohol drinking, smoking, physical activities and/or hobbies, and coffee and black tea consumption.

## Discussion

This prospective longitudinal study demonstrated that daily green tea consumption is significantly associated with a decreased risk of cognitive decline (dementia or MCI), even after controlling for potential confounding factors. In addition, higher green tea consumption was inversely associated with dementia in both age- and sex- adjusted models. To the best of our knowledge, this is the first longitudinal study that examined the association between green tea and the incidence of dementia and cognitive decline. In a cross-sectional study, higher green tea consumption was associated with a lower prevalence of cognitive impairment [16]. In that study, although cognitive function was assessed with MMSE, the incidence of dementia or MCI was not investigated.

In the present study, no relationship was found between coffee consumption and a risk of dementia or cognitive decline. In contrast, higher coffee consumption was reported to decrease the risk of AD over a 5-year period [10] and was associated with a decrease in cognitive decline in a 10-year follow-up [11]. Furthermore, in the Three City Study [12], consuming more than three cups of a caffeinated beverage (coffee or black tea) per day was associated with a lower decline in cognitive tests among elderly women, but there was no relationship between caffeine consumption and dementia risk over a 4-year period, similar to our results. The relatively short follow-up period and the small sample size might be reasons why we did not observe protective effects of coffee against dementia or cognitive decline.

In the present study and in previous longitudinal studies [14], [15], no association was found between black tea consumption and the risk of dementia or cognitive decline. Drinking black tea was a relatively uncommon practice in the area of our study, which may have resulted in low statistical power.

Both green and black teas contain polyphenols, caffeine, L-theanine, and other nutrients. The major tea-related polyphenols present in green tea are catechins, especially epigallo catechin 3-gallate (EGCG), whereas black tea mainly contains theaflavins [27]. In addition, green tea contains more myricetin compared with black tea [27]. Other tea-related polyphenols such as quercetin, kaempferol, apigenin, and luteolin, are also present in both green and black tea, but the amounts of these polyphenols are not significantly different between tea types [27]. EGCG is permeable to the blood brain barrier [28] and exerts neuroprotective and neurorescue effects against amyloid β (Aβ) toxicity by inhibiting Aβ aggregation [29] and production [3], [29]. Myricetin inhibits Aβ aggregation, especially oligomerization *in vitro* [4], [30]. Furthermore, oral administration of EGCG or myricetin prevents the development of AD pathology in AD-model mice [5], [6], [31]. The positive relationships observed in this study between green tea consumption and both dementia and cognitive decline, and the null relationship between black tea consumption and either dementia or cognitive decline may support previously reported data [1]–[6], [29]–[31] regarding significant neuroprotective effects of EGCG and myricetin.

The caffeine content is 40–57 mg/100 mL in coffee [32], 25.5 mg/100 mL in black tea, and only 15.3 mg/100 mL in green tea [33]. Caffeine is a nonselective A1 and A2a adenosine receptor antagonist that stimulates cholinergic neurons [8]. Chronic caffeine administration was shown to have neuroprotective effects in a mouse model of AD, indicating that decreased Aβ production is a likely mechanism [8]. Moreover, both caffeine and adenosine A2a receptor antagonists prevent Aβ-induced cognitive deficits in mice [9]. Our study suggests that the contribution of caffeine to cognitive function may be small due to the null relationship observed between coffee consumption and cognitive impairment.

High intake of ascorbic acid is associated with lower risk of AD [34]. The content of ascorbic acid is 6 mg/100 mL in green tea, which is the most common source of ascorbic acid in Japan [35], [36]. On the other hand, coffee and black tea do not contain ascorbic acid [36]. We cannot exclude the possibility that ascorbic acid in green tea had a positive effect on cognitive function. However, this explanation was not supported by the findings that the serum levels of ascorbic acid were associated with the frequency of coffee consumption, but not green tea consumption, thereby indicating that the effects of ascorbic acid on cognitive function may be small. L-theanine, an amino acid rich in green and black teas, but not present in coffee, has antioxidative properties and neuroprotective effects through inhibition of both Aβ-induced oxidative stress and activation of the ERK1/p38 MAPK and NK-kB pathways [37]. Further studies that determine L-theanine levels in participants are required.

We could not fully exclude the possibility that the analyses were confounded by unmeasured factors. For example, green tea consumption is associated a variety of health behavior or social factors, and previous studies have shown that physical activity and hobbies are associated with lower risk of dementia and MCI [38], [39]. Hence, the association of more frequent consumption of green tea with more physical activities and hobbies reported here may not be surprising. However, we found that the inverse association between green tea consumption and incidence of dementia and cognitive decline was present even after adjustment for physical activities and hobbies. All participants were free from cognitive impairment and had at least MMSE score of 24 or higher at the time of the baseline survey. However, as lower MMSE scores were associated with smaller amounts of green tea consumption, participants who consumed smaller amounts of green tea might have had very mild cognitive impairment at baseline.

There are some limitations to the present study. First, because the sample size was relatively small, we did not assess the association of the combined frequency of green tea, coffee, and black tea consumption with incidence of dementia or cognitive decline. Second, because we did not include the question about the amounts of cups of beverage consumption at baseline survey, we could not assess the association between amounts of beverage consumption and cognitive decline. Third, we did not evaluate the causes of dementia and MCI with diagnostic tools such as neuroimaging and neuropathology. Further studies involving neuroimaging and neuropathology are required to reveal the cause of dementia and MCI in this population and to evaluate the effects of green tea consumption for each cause of dementia. Furthermore, among the number of subjects at baseline (n = 723), the valid response rate (67.8%, n = 490) was not high. The strengths of the current study are its longitudinal design and the opportunity to adjust for possible confounding factors.

In conclusion, higher green tea consumption was associated with lower incidence of cognitive decline (dementia or MCI) in an elderly Japanese population. Our results suggest that green tea consumption could be beneficial for reducing the risk of cognitive decline.

## Supporting Information

Table S1 **Characteristics of the participants including the final analysis and subjects lost to follow-up.** Values expressed as mean (SD) unless otherwise indicated. MMSE: Mini-mental state examination.
(DOC)
