# Supplementary material for: Consumption of Green Tea, but Not Black Tea or Coffee, Is Associated with Reduced Risk of Cognitive Decline
Source: PLoS One. 2014 May 14;9(5):e96013. doi: 10.1371/journal.pone.0096013 (PMC4020750; doi:10.1371/journal.pone.0096013)
Supplement: Table S1 — Characteristics of the participants including the final analysis and subjects lost to follow-up. Values expressed as mean (SD) unless otherwise indicated. MMSE: Mini-mental state examination. (DOC) [file pone.0096013.s001.doc]

Table S1

| Number of participants | Participants of final analysis  n = 490 | Subjects lost to follow-up  n = 167 | P-Value |
| --- | --- | --- | --- |
| Age at baseline survey, years | 71.2 (6.4) | 70.5 (6.8) | 0.247 |
| Sex: women, % | 66.9 | 61.7 | 0.221 |
| Education, years | 9.9 (2.2) | 9.9 (2.4) | 0.915 |
| MMSE, points, Median (SE) | 28.0 (0.1) | 28.0 (0.1) | 0.329 |
| ApoE E4 carriers, % | 21.8 | 19.9 | 0.657 |
| Hypertension at baseline, % | 44.3 | 45.5 | 0.788 |
| Hyperlipidemia at baseline, % | 17.1 | 18.0 | 0.814 |
| Diabetes mellitus at baseline, % | 12.7 | 13.2 | 0.893 |
| Smokers (Current), % | 10.8 | 10.8 | 1.000 |
| Alcohol drinkers (Current), % | 37.8 | 38.3 | 0.927 |
| Physical activities/ hobbies (Current), % | 75.5 | 77.2 | 0.676 |
| Green tea consumption, 1-6 days/ every day, % | 39.8/ 32.0 | 46.1/ 26.9 | 0.314 |
| Coffee consumption, 1-6 days/ every day, % | 36.7/ 43.3 | 35.9/ 41.9 | 0.838 |
| Black tea consumption, 1-7 days/ week, % | 17.6 | 15.6 | 0.634 |
